# IgG3 enhances neutralization potency and Fc effector function of an HIV V2-specific broadly neutralizing antibody

**DOI:** 10.1371/journal.ppat.1008064

**Published:** 2019-12-16

**Authors:** Simone I. Richardson, Bronwen E. Lambson, Andrew R. Crowley, Arman Bashirova, Cathrine Scheepers, Nigel Garrett, Salim Abdool Karim, Nonhlanhla N. Mkhize, Mary Carrington, Margaret E. Ackerman, Penny L. Moore, Lynn Morris

**Affiliations:** 1 Centre for HIV and STI’s, National Institute for Communicable Diseases, Johannesburg, Gauteng, South Africa; 2 Faculty of Health Sciences, University of the Witwatersrand, Johannesburg, Gauteng, South Africa; 3 Thayer School of Engineering, Dartmouth College, Hanover, New Hampshire, United States of America; 4 Ragon Institute of Massachusetts General Hospital, MIT, and Harvard University, Boston, Massachusetts, United States of America; 5 Basic Science Program, Frederick National Laboratory for Cancer Research, Frederick, Maryland, United States of America; 6 Centre for the AIDS Programme of Research in South Africa (CAPRISA), University of KwaZulu-Natal, Congella, KwaZulu-Natal, South Africa; 7 Department of Public Health Medicine, School of Nursing and Public Health, University of KwaZulu-Natal, Durban, South Africa; 8 Department of Epidemiology, Columbia University, New York, NY, United States of America; King's College London, UNITED KINGDOM

## Abstract

Broadly neutralizing antibodies (bNAbs) protect against HIV infection in non-human primates and their efficacy may be enhanced through interaction with Fc receptors on immune cells. Antibody isotype is a modulator of this binding with the IgG3 subclass mediating potent Fc effector function and is associated with HIV vaccine efficacy and HIV control. BNAb functions are typically assessed independently of the constant region with which they are naturally expressed. To examine the role of natural isotype in the context of a bNAb lineage we studied CAP256, an HIV-infected individual that mounted a potent V2-specific bNAb response. CAP256 expressed persistently high levels of plasma IgG3 which we found mediated both broad neutralizing activity and potent Fc function. Sequencing of germline DNA and the constant regions of V2-directed bNAbs from this donor revealed the expression of a novel *IGHG3* allele as well as IGHG3*17, an allele that produces IgG3 antibodies with increased plasma half-life. Both allelic variants were used to generate CAP256-VRC26.25 and CAP256-VRC26.29 IgG3 bNAbs and these were compared to IgG1 versions. IgG3 variants were shown to have significantly higher phagocytosis and trogocytosis compared to IgG1 versions, which corresponded to increased affinity for FcγRIIa. Neutralization potency was also significantly higher for IgG3 bNAbs, particularly against viruses lacking the N160 glycan. By exchanging hinge regions between subclass variants, we showed that hinge length modulated both neutralization potency and Fc function. This study showed that co-operation between the variable and natural IgG3 constant regions enhanced the polyfunctionality of antibodies, indicating the value of leveraging genetic variation which could be exploited for passive immunity.

## Author summary

In the only partially effective HIV vaccine trial to date, reduced risk of infection correlated with IgG3 antibodies that bound to the V2 region of the HIV envelope. IgG3 antibodies potently mediate the broadest range of Fc cytotoxic functions such as complement deposition, cellular lysis, engulfment and membrane nibbling. The relevance of different IgG3 alleles in antibody function is not typically examined, nor are antibodies usually assessed in the context of their natural isotype. Here we show that IgG3 V2-specific broadly neutralizing antibodies from donor CAP256 mediated both enhanced cytotoxic functions and neutralization potency compared to an IgG1 variant. We demonstrate that the long hinge region of IgG3 facilitated this increased functionality. These data suggest that isotype may be tuned to enhance both neutralization and cytotoxic activity of broadly neutralizing antibodies that can be used to improve the efficacy of passive immunization strategies for HIV prevention.

## Introduction

Antibodies are multifunctional molecules that mediate both pathogen neutralization by the Fab-antigen interaction and a variety of Fc effector functions through their ability to engage cellular receptors. Passive transfer of HIV-specific broadly neutralizing antibodies (bNAbs) has been shown to provide sterilizing immunity in animal models and their induction therefore remains a major goal of vaccination [1]. However, accumulating evidence suggests that Fc effector functions also contribute to the protection afforded by these bNAbs [2–5]. Fc-dependent mechanisms can restrict the number of transmitted/founder viruses that establish infection, impact viral load, drive viral escape [6–9] and are associated with HIV control [10] and slower disease progression [11, 12]. In the partially effective RV144 HIV vaccine trial, reduced risk of infection correlated with Fc effector function mediated largely by IgG3 antibodies directed to the V1V2 region of HIV envelope [13, 14]. These included antibody-dependent cellular cytotoxicity (ADCC) or cell lysis, cellular phagocytosis (ADCP) and complement deposition [13–16] illustrating for the first time the importance of Fc functions and their modulators, such as isotype, in HIV prevention.

Despite the antibody Fc being highly conserved, variation in the constant regions of the heavy chain (CH1-3) genes produces different isotypes (IgM, IgA, IgG and IgE) and subclasses (IgG1-4 and IgA1-2) as well as allelic variants. These isotypes have varied structures, immune complex formation, half-life and binding affinities for Fc receptors, which allows them to engage differentially with a variety of cells and modulate Fc effector functions [17]. There is now substantial evidence that different isotypes and their polymorphisms show varying protection in a number of diseases including HIV, although a mechanism for this is not often explored [18–21].

IgG3 is the most polyfunctional of the IgG subclasses, eliciting the widest range and most potent Fc effector functions [22]. This isotype typically appears early in HIV infection [23, 24] and has been shown to decrease with disease progression coinciding with the reduction in Fc effector functions [25]. Despite occurring at low titers, IgG3 antibodies are potent mediators of HIV vaccine induced responses [13, 14, 26] and are associated with HIV control in a number of cohorts [10, 27, 28], suggesting that IgG3 maintenance could be beneficial for prevention. However, a recent study has shown that IgG3 in chronic infection can dampen B cell responses [29], highlighting the complex and multiple roles of IgG3 in HIV infection. The *IGHG3* gene is highly polymorphic with 29 reported alleles [30], providing another layer of variability, though the functional relevance of this is largely unknown. Although most IgG3 variants are associated with shorter half-lives in plasma compared to IgG1 [23, 24], five IgG3 allelic variants have an half-life equivalent to IgG1 [31]. Furthermore, depending on the allelic variant, the IgG3 hinge linking the Fab and the Fc regions is 2 to 4 times longer than IgG1. This increased hinge length can affect antibody stability, flexibility and antigen affinity which in turn impacts on function and may translate to differential protection [32–34]. There is therefore strong evidence that allelic variation in IgG3 could directly impact on Fc effector function and neutralization mediated by the distal Fab.

In this study, we aimed to examine whether the function of a bNAb can be improved when expressed as an IgG3 as well as to explore the value of studying antibodies as they are naturally expressed. We previously described CAP256, an HIV-infected donor that developed a potent V2 directed broadly neutralizing response and maintained high levels of HIV-specific IgG3 over 3 years [35]. Here, we show that the IgG3 plasma response in CAP256 contributes to both neutralization breadth and Fc function. Further, we describe CAP256-VRC26 V2 bNAb lineage members [36, 37] that use a novel *IGHG3* allele and the presence of a long-lived IGHG3*17 allele which confers improved Fc effector function and neutralization potency. This study provides the first example of the IgG3 subclass modulating improved neutralization potency by a V2-specific bNAb, and defines a novel mechanism through which bNAbs could be engineered for enhanced potency and protection.

## Results

### IgG3 mediates Fc effector functions and neutralization in donor CAP256

**Fig 1:**
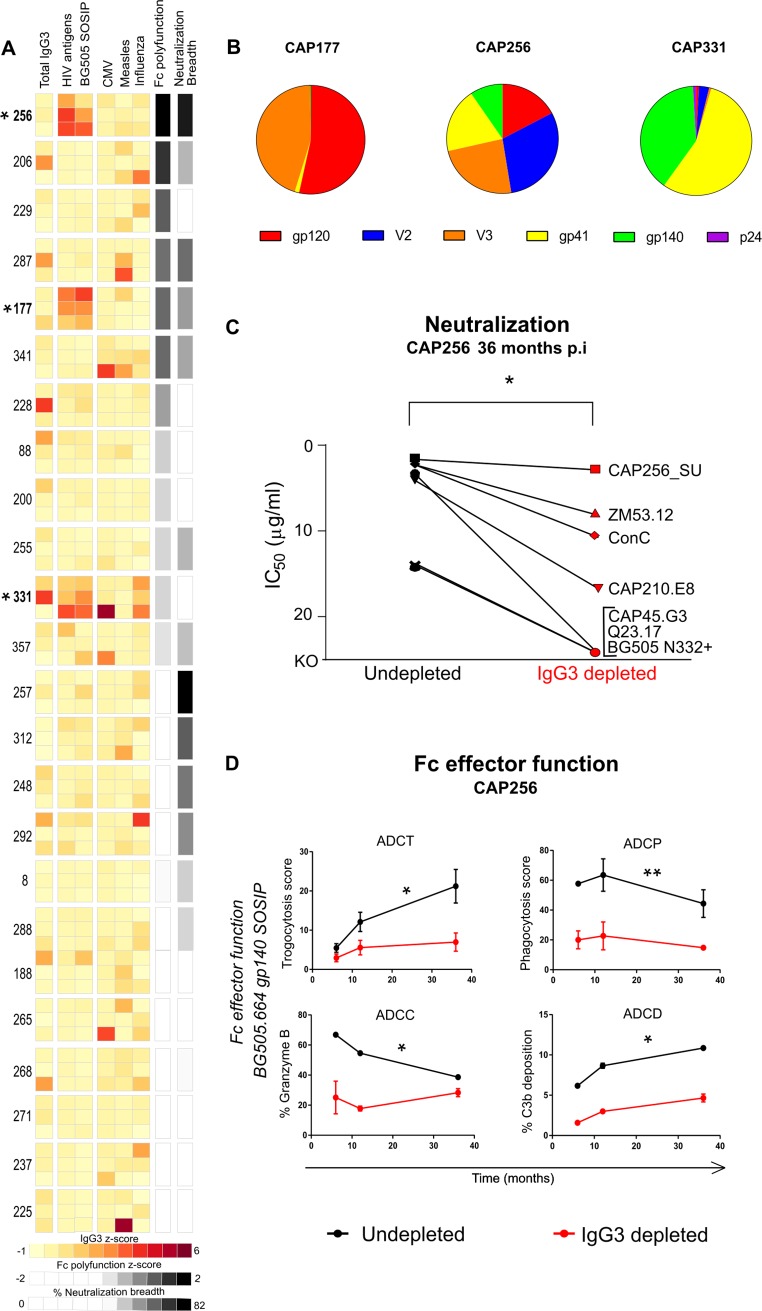
HIV specific IgG3 mediates both Fc effector functions and neutralization potency in CAP256. **(A)** Total and antigen-specific IgG3 levels (against 14 HIV antigens, BG505.SOSIP.664, cytomegalovirus glycoprotein B, measles nucleoprotein and influenza hemagglutinin) were measured in plasma IgG from 24 CAPRISA individuals by ELISA or multiplex assay. Data for 6, 12 and 36 months p.i. for each individual are stacked, represented as Z-scores of IgG3 normalized to total IgG with an asterisk indicating individuals with high HIV-specific IgG3 levels. Data is ranked by total Fc polyfunctionality Z-scores and shown on a grey scale defined as the addition of ConC gp120 specific ADCC (% granzyme B in live target CEM.NKR.CCR5 cells), ADCP (% THP-1 cells that take up fluorescent beads x MFI), ADCT (relative proportion of PKH26 transferred from target cells to CSFE+ THP-1 cells) and ADCD (% C3b deposition) over time. Neutralization breadth against a 44 virus panel at 36 months p.i. is shown in grey shading. **(B)** The relative proportion of IgG3 antibodies binding to 14 different HIV-specific antigens and grouped by epitope are shown for CAP177, CAP256 and CAP331 at 36 months p.i. **(C)** Neutralization potency (IC_50_ in μg/ml) of undepleted (black) and IgG3 depleted (red) polyclonal IgG from CAP256 at 36 months p.i. against multiple viruses where KO = knock-out or greater that 25μg/ml. Significant difference is indicated as *p<0.05 determined by a Wilcoxon matched-pairs signed rank test. **(D)** Fc effector function against BG505.SOSIP.664 for CAP256 IgG3 depleted (red) and undepleted (black) samples. Significant differences shown as *p<0.05 and **p<0.01 were determined by Friedman’s test with Dunn’s correction. All data are representative of three independent experiments.

Using a custom IgG subclass multiplex assay [38] with 14 different HIV antigens as well as a BG505.SOSIP.664 trimer ELISA, we screened samples from 24 HIV-infected donors in the CAPRISA 002 acute infection cohort [39, 40] at 6, 12 and 36 months post infection. Only three donors were found to have high levels of HIV-specific IgG3 (highlighted with an asterisk in Fig 1A). In donor CAP177, these levels decreased over time, which is typical of HIV infection [23] while donor CAP331 had an IgG3 response to multiple non-HIV antigens, suggesting a general IgG3-skewed humoral response unique to this individual. As observed in a previous study, we confirmed that donor CAP256 had high levels of HIV-specific IgG3 that increased over time [35], with little reactivity to non-HIV antigens. CAP256 showed the highest neutralization breadth and Fc polyfunctionality Z-score of all participants indicated for 36 months post infection (Fig 1A). Additionally, the HIV-specific IgG3 plasma antibodies in CAP256 targeted a broader range of HIV antigens compared to CAP177 and CAP331 (Fig 1B).

To determine whether the IgG3 antibodies in donor CAP256 had functional activity, we depleted IgG3 from total plasma IgG using anti-IgG3 magnetic beads [13] at 36 months post-infection, the time point of peak neutralization breadth. Treated plasma IgG was confirmed to be IgG3-depleted by ELISA and tested against a panel of seven sensitive viruses. Except for the autologous CAP256_SU virus, there was a significant decrease in neutralization titre (p = 0.016; Wilcoxon matched-pairs signed rank test) following IgG3-depletion as indicated by a greater than 3-fold change or knock-out (>25 μg/ml) compared to undepleted samples (Fig 1C, S1 Fig). While residual neutralization following depletion demonstrates that other IgG subclasses also contribute, a large proportion of the neutralization response against these viruses was mediated by IgG3 mAbs.

We next examined Fc effector function using IgG3-depleted samples from 6, 12 and 36 months post-infection against BG505.SOSIP.664 trimer. These functions included antibody-dependent cellular phagocytosis (ADCP) measured as the engulfment of coated beads by a monocytic cell line [41], antibody dependent cellular trogocytosis (ADCT) measured as the transfer of PKH26 membrane dye from antigen coated target cells to THP-1 cells [42], antibody-dependent cellular cytotoxicity (ADCC) indicated as the percentage of granzyme B secreted by NK cells on to antigen coated target cells [43] and finally antibody-dependent complement deposition (ADCD) determined by the deposition of C3b on the surface of antigen coated cells [10]. Significant decreases in ADCT, ADCP, ADCC and ADCD were observed for CAP256 IgG3-depleted samples (Fig 1D). These data indicate that the persistent HIV-specific IgG3 response in plasma from donor CAP256 mediates both potent neutralization and multiple Fc effector functions.

### A bNAb isolated from CAP256 uses a novel *IGHG3* allele

**Fig 2:**
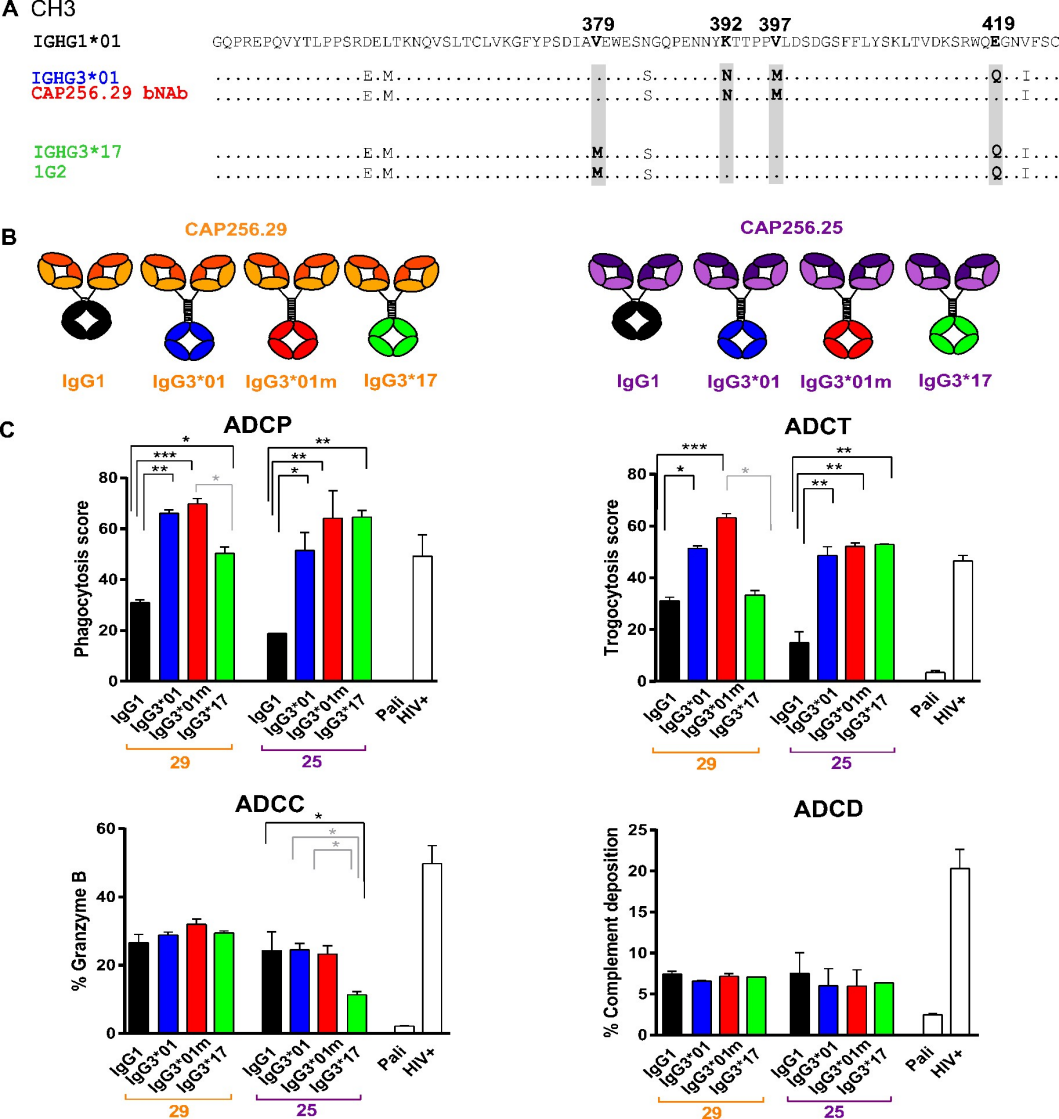
IgG3 allelic variants of CAP256 bNAbs show enhanced ADCP and ADCT. **(A)** Amino acid sequences of the CH3 region of antibodies isolated from CAP256 aligned to *IGHG1*. Alignment of IGHG3*01 (blue), novel allele designated IgG3*01m from CAP256.29 bNAb (red), non-HIV mAb CAP256.1G2 and IGHG3*17 (green). SNPs that differ between IgG3 variants are indicated in grey. **(B)** CAP256.29 (orange) and CAP256.25 (purple) variants were expressed as IgG1, IgG3*01, *01m and *17. **(C)** All variants of CAP256.29 and CAP256.25 were tested for ADCP (% THP-1 cells that take up antigen coated fluorescent beads x MFI), ADCT (relative proportion of PKH26 transferred from target cells to CSFE+ THP-1 cells), ADCC (% granzyme B in live target cells) and ADCD (% C3b deposition on the surface of target cells) against BG505.SOSIP.664. gp140 trimer coated CEM.NKR.CCR5 target cells. Palivizumab (Pali) was a negative control with HIV+ pooled polyclonal IgG as a positive control. Significant differences between IgG1 and IgG3 variants are indicated in black and between IgG3 allelic variants in grey. For all experiments statistical significance was calculated using Friedman’s test with Dunn’s correction for multiple comparisons where *p<0.05, **<0.01 and ***<0.001. Mean and standard deviation of 3 independent experiments are represented.

We previously isolated a large family of bNAbs from donor CAP256 that target a quaternary V2 epitope present only on the HIV trimer [36, 37]. Using remnant cDNA from previous B cell sorts, we amplified the entire CH1-CH3 and hinge regions of CAP256-VRC26.29 (referred to hereafter as CAP256.29) (Fig 2A and S2 Fig). Sequence analysis revealed that this bNAb utilised a previously undescribed *IGHG3* allele, which we designated IGHG3*01m (GenBank identifier: MK679684). This novel allele differs from IGHG3*01 at position 419 and from IGHG3*13 at position 392 (S3 Fig). Two additional bNAbs from this lineage (CAP256.30 and CAP256.33) were also found to belong to the IgG3 subclass (S2 Fig). The sequence of the CH1-3 region from CAP256 genomic DNA was consistent with the IGHG3*01m/17 genotype (S2 Fig). This observation was further confirmed by sequencing cDNA from the mAb 1G2 and three other unrelated heavy chains isolated from the same donor that were distinct from the CAP256-VRC26 bNAb lineage and used the IGHG3*17 allele (Fig 2A and S2 Fig). This allele is of particular interest as it has a half-life equivalent to IgG1, unlike the majority of other IgG3 variants which have significantly shorter half-lives as a result of their lower affinity for the Fc neonatal receptor attributed to an arginine at position 435 [31].

### CAP256 bNAbs expressed as IgG3 show improved Fc effector function

To investigate the functional impact of these different constant regions, we generated CAP256.29 and CAP256.25 (a more potent and broad lineage member) as IgG3*01, IgG3*01m and IgG3*17 variants (Fig 2B). The purity of these preparations was assessed by native PAGE with minimal evidence of dimerization for both subclasses. As expected all IgG3 variants were approximately 10kDa larger than IgG1 variants.

These antibodies were tested for Fc effector function activity against BG505.SOSIP.664 trimer, with concentrations tailored to take into account the varying size of the proteins. All IgG3 variants of both CAP256.29 and CAP256.25 showed significantly higher levels of ADCP and ADCT than IgG1 variants (represented at the concentration of peak activity in Fig 2C and over a range of concentrations in S4 Fig), in line with a number of studies that have shown enhanced phagocytosis by IgG3 antibodies [44–46] (Chu, co-submission). However, there was no improvement in ADCC or ADCD, with both CAP256.25 and CAP256.29 bNAbs generally showing low ADCD levels compared to a polyclonal HIV positive control. In several cases there were significant differences between allelic variants of IgG3 in their ability to mediate ADCP, ADCT and ADCC (shown by grey significance bars) indicating that not only subclass but allelic variation can affect some Fc-mediated effector functions.

### Enhanced Fc effector function tracks with Fc receptor interaction and not increased antigen binding

**Fig 3:**
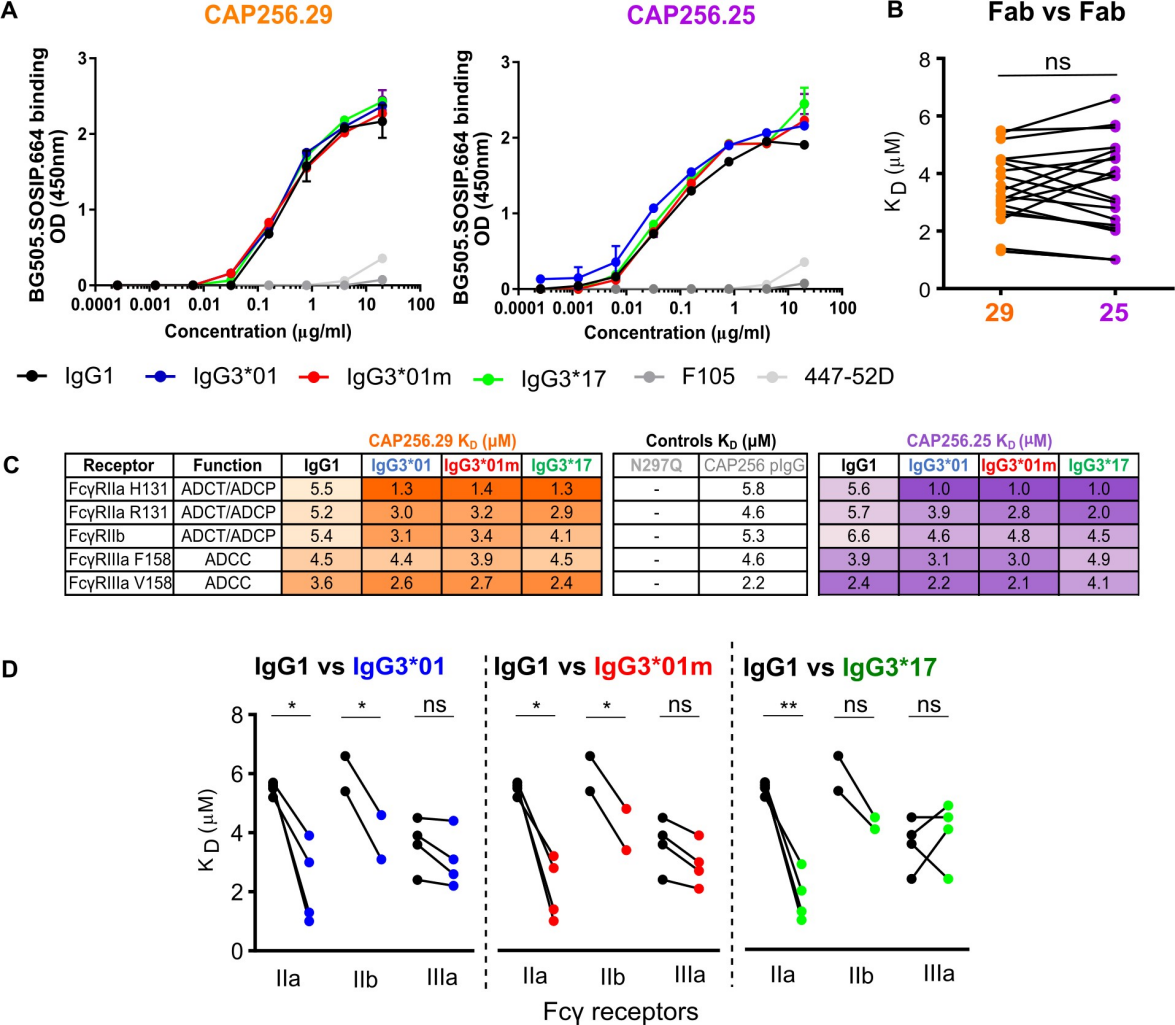
Fcγ receptor and not antigen binding is associated with differential Fc effector function. **(A)** Binding to BG505.SOSIP.664 trimer by ELISA is shown for all variants of CAP256/29 and CAP256.25 respectively. F105 and 447-52D are shown as negative controls. Plots are representative of 2 independent experiments. **(B)** Dissociation equilibrium constants (K_D_ in μM) determined by surface plasmon resonance (SPR) of subclass variants for all Fc receptors are grouped by bNAb. Significance was determined by paired t test with ns = non-significant. **(C)** Dissociation equilibrium constants for all variants of CAP256.29 and CAP256.25 binding to 5 different Fc receptors. Linked points indicate antibodies with the same Fc portion binding to the same Fc receptor but with either CAP256.29 (orange) or CAP256.25 (purple) Fab portions. CAP256 polyclonal IgG was a positive control and VRC01 N297Q was a negative control. Data are representative of 2 independent experiments. **(D)** Dissociation equilibrium constants (K_D_ in μM) are shown for IgG1 compared to IgG3 variants for each of the Fc receptors tested. Polymorphic variants of each receptor as well as affinities for both CAP256.29 and CAP256.25 are grouped together. Significance is indicated as *p<0.05 or **p<0.001 and was determined by paired t test.

To determine if Fc effector function was dependent on antigen affinity, we assessed if IgG3 variants exhibited differences in binding to BG505.SOSIP.664 trimer. Fc effector functions have been previously shown to be affected by the affinity of the Fab for antigen [47]. Using an anti-Fab ELISA, to account for potential isotypic bias of anti-IgG antibodies, we found no difference in affinity of CAP256 antibodies for the trimer regardless of subclass (Fig 3A and 3B).

We next examined the affinity of the antibody for Fc receptors which varies by subclass and is known to modulate Fc effector function. A previous study reported enhanced binding of IgG3 antibodies to different FcɣRs [17]. We therefore used surface plasmon resonance (SPR) to investigate whether the increased Fc effector function mediated by IgG3 tracked with stronger binding of the variants to relevant low affinity Fc receptors. CAP256.29 and CAP256.25 antibodies were assessed for strength of binding to FcγRIIa and FcγRIIb, which mediate ADCP and ADCT, and FcγRIIIa, required for ADCC [48]. Polymorphic variants of Fc receptors known to affect affinity for IgG including FcγRIIa (R131 vs H131) and FcγRIIIa (F158 vs V158) were also tested [17]. Micromolar K_D_ values are shown in Fig 3B–3D with representative kinetic curves and standard deviation of affinities shown in S5 Fig. Differences in Fc receptor affinity between the same Fc variants of CAP256.29 and CAP256.25 were not significant overall (Fig 3B) as expected, given that the Fab is unlikely to play a role in Fc receptor binding in the absence of antigen. In agreement with the observed increases in ADCP and ADCT activity, the IgG3 versions of both CAP256.29 and CAP256.25 showed moderate but significantly stronger binding affinities to FcγRIIa and FcγRIIb compared to IgG1 (Fig 3C and 3D). Most pronounced was the 3.7–5 fold enhancement of IgG3 variants for FcγRIIA H131 binding compared to IgG1. FcγRIIIa binding was similar for all IgG3 variants and IgG1 (Fig 3D), consistent with the comparable levels of ADCC activity, except for CAP256.25 IgG3*17 which showed the weakest binding (ranging between 1.7–1.9 fold less than other variants) and lowest levels of ADCC (Fig 2C). Overall, greater affinity for FcγRIIa/b tracked with enhanced ADCP and ADCT function.

### Allelic variation in the CH3 of IgG3 alters ADCC activity

The lower levels of ADCC activity by CAP256.25 IgG3*17 compared to the other IgG3 allelic variants was unexpected (Fig 2C and 2D). Analysis of the sequences revealed that IgG3*17 has a lysine at position 392 (Fig 2A) that abrogates a potential N linked glycan (PNG) motif important for stability [33] and has been previously associated with differences in ADCC [49]. To explore this, we mutated the lysine to an asparagine to match IgG3*01m (Fig 2A, S2 Fig). Restoring this PNG motif resulted in a significant increase in ADCC activity but had no impact on other functions (S6 Fig). This finding was supported by a concomitant increase in the affinity of the mutant for both polymorphic variants of FcγRIIIa (S6 Fig). The K392N mutant also showed significantly increased binding to FcγRIIb, but did not impact ADCP activity, possibly because affinity for the FcγRIIa receptors remained unchanged. These data further illustrate the relationship between Fc receptor binding and Fc effector function and indicate that allelic variation in CH3 can impact significantly on Fc activity.

### IgG3 variants of CAP256 bNAbs show significantly higher neutralization potency

**Fig 4:**
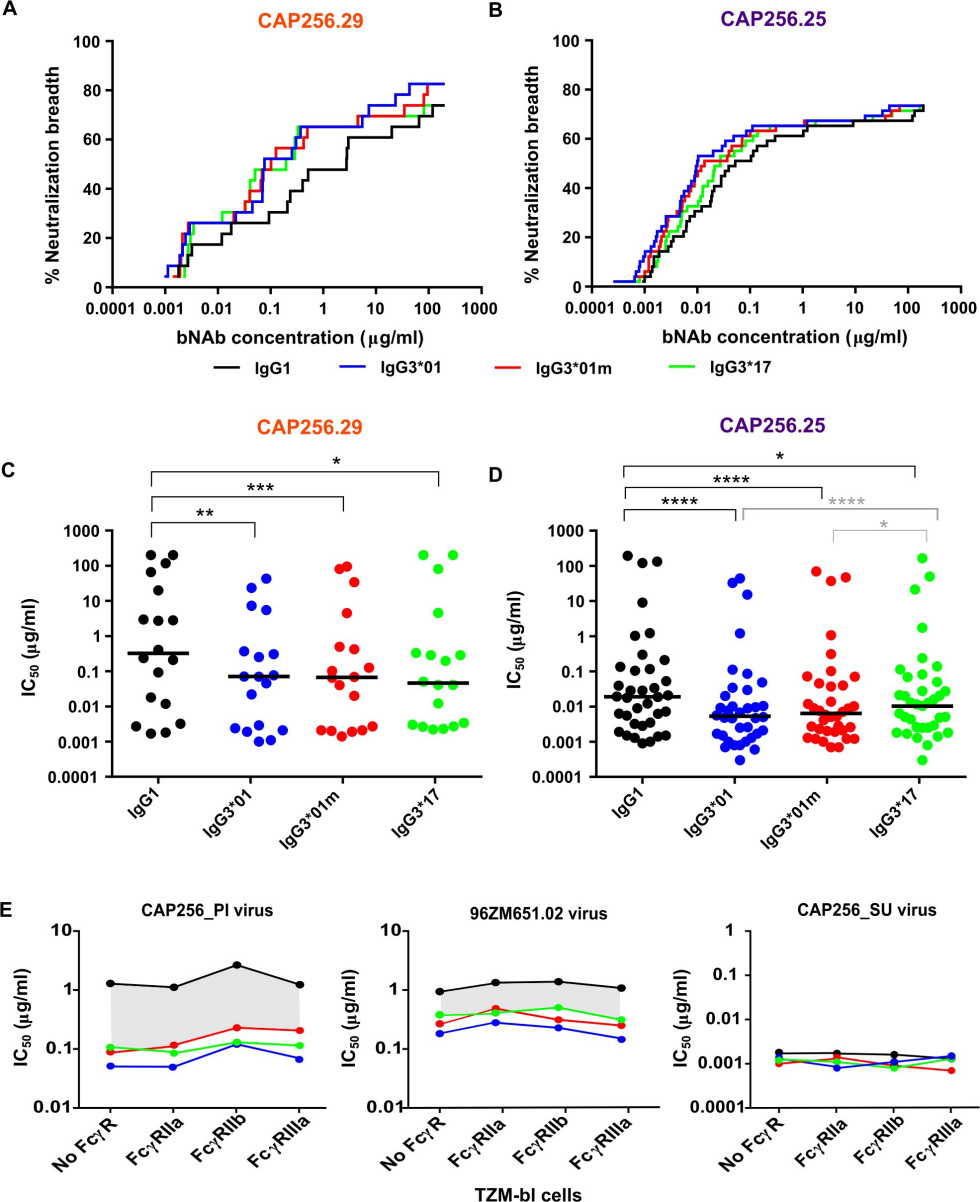
IgG3 variants of CAP256.29 and CAP256.25 show enhanced neutralization compared to IgG1. Neutralization breadth-potency curves for IgG1 (black), IgG3*01m (red), IgG3*01 (blue) and IgG3*17 (green) variants of **(A)** CAP256.29 tested against a 22 virus panel and **(B)** CAP256.25 tested against a 49 virus panel. Median IC_50_ of all variants of **(C)** CAP256.29 and **(D)** CAP256.25 bNAbs are shown with significant differences calculated by Friedman test with Dunn’s test for multiple corrections where ****p<0.0001, ***p<0.001, **p<0.01, *p<0.05. Differences between IgG1 and IgG3 variants are shown in black and those between IgG3 allelic variants in grey. Values are representative of 3 independent experiments. **(E)** IC_50_ of CAP256.25 variants against viruses CAP256_PI, 96ZM651.02 and CAP256_SU are shown using TZM-bl cells without Fc receptors (WT) and those expressing FcγRI, FcγRIIa, FcγRIIb or FcγRIIIa. The grey area indicates the difference between neutralization potencies of the IgG1 and IgG3 variants.

We next tested whether the use of an IgG3 constant region affected neutralizing activity of the CAP256 antibodies using a large panel of viruses in the robust TZM-bl assay. CAP256.25 is significantly more broad than CAP256.29 and so a well-characterised 49 virus panel and a 22 virus panel were used for each mAb respectively (S1 Table). Assays were performed at least 3 times in a head-to-head comparison with the operator blinded to the antibody variant being tested. All IgG3 variants showed significantly enhanced potency over IgG1 for both CAP256.29 (Fig 4A and 4C) and CAP256.25 (Fig 4B and 4D) overall. IgG3 variants of CAP256.29 also showed a slight increase in breadth over IgG1 (83% vs 74%, S1 Table). In addition, there were significant differences between CAP256.25 IgG3 allelic variants, with IgG3*01 and IgG3*01m showing increased potency compared to IgG3*17 (Fig 4C). This effect was not common to all viruses (S1 Table), suggesting a contributing role for a viral signature, The lack of differential ELISA binding for BG505.SOSIP.664 by the subclass variants (Fig 3A) was mirrored by neutralization where there was no fold-change between IgG3 and IgG1 bNAbs against the BG505 pseudovirus (S1 Table).

Given that IgG3 subclass variants have higher affinities for Fc receptors and neutralization by bNAbs has previously been shown to be enhanced by the presence of Fcγ receptors [50], we aimed to assess if neutralization subclass differences were amplified in the presence of these receptors. We therefore tested neutralization using TZM-bl cells transduced with one of three Fc receptors (FcγRIIa, FcγRIIb and FcγRIIIa). There were no significant increases in neutralization titres of IgG1 or any IgG3 allelic variants compared to standard TZM-bl cells that lack Fc receptors (grey area is constant across cell types, Fig 4E). This indicated that neutralization differences between subclasses was unaffected by the presence of Fc receptors.

### Viral signature of IgG3 neutralization enhancement

**Fig 5:**
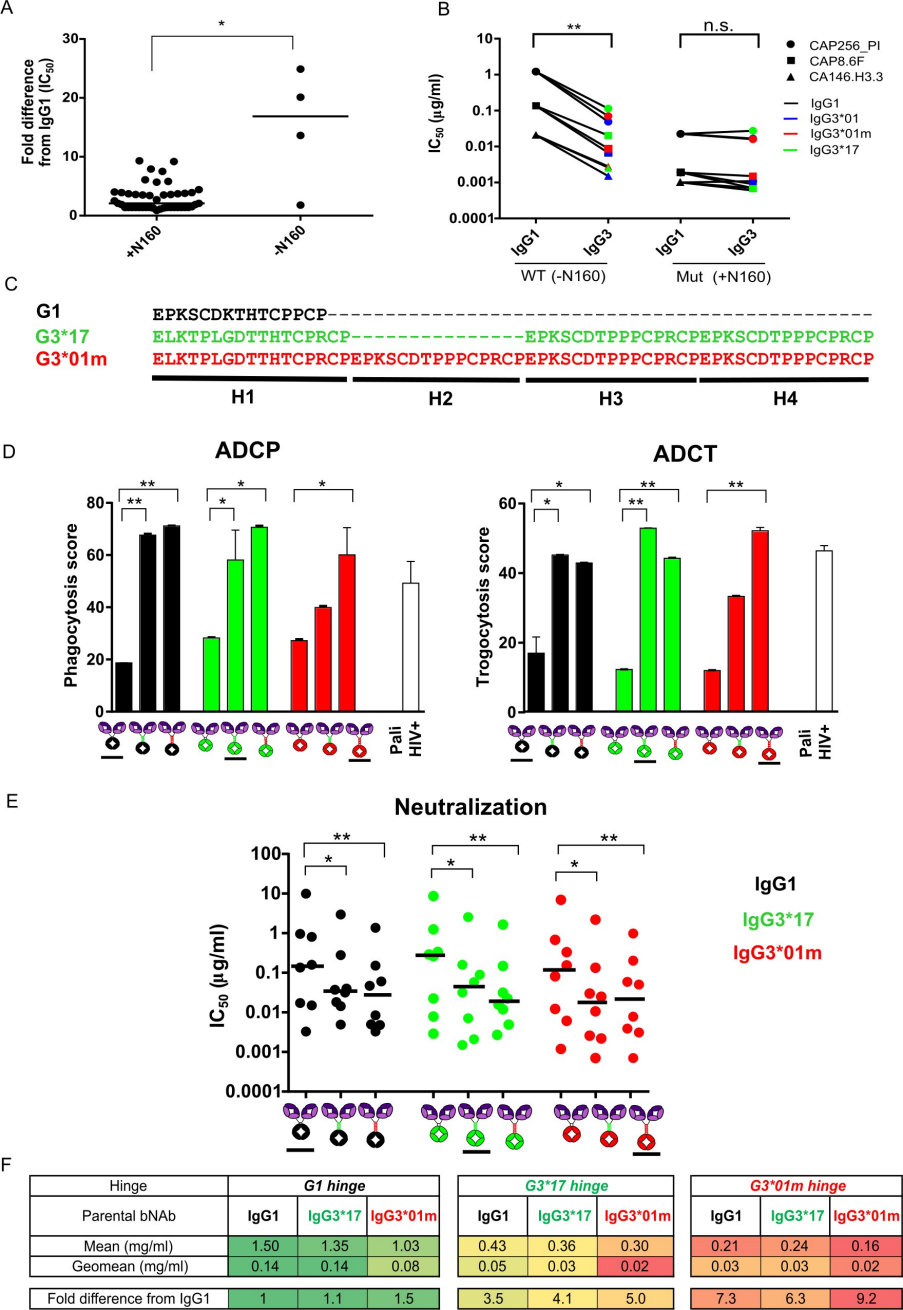
Viral and hinge length mechanisms of enhanced Fc effector function and neutralization potency of IgG3 bNAbs. **(A)** Fold-difference in IC_50_ neutralization titres between IgG1 and IgG3*01 versions of CAP256.25 among 36 viruses with/without a potential PNG at position 160. Significance was calculated using a Mann-Whitney test *p<0.05. **(B)** Neutralization titres of IgG1 and all IgG3 allelic variants against 3 viruses (indicated by shape) that lacked the PNG motif at 160 (-N160) and 160 knock-in mutants (+N160). Significant differences calculated by the Wilcoxon sign-ranked test where **p<0.01 are shown. **(C)** Hinge sequences of IgG1, IgG3*01m and IgG3*17 are shown with hinge exons H1-H4 indicated. **(D)** ADCP and ADCT activity of CAP256.25 hinge variants and wild-type IgG1 or IgG3 variants (underlined) against BG505.SOSIP.664. Palivizumab is used as a negative control with a clade C HIV+ pool used as a positive control. Mean and standard deviations are indicated with significance calculated using the Friedman test with Dunns correction for multiple comparisons which is shown by *p<0.05 and **p<0.01. **(E)** Neutralization titres of CAP256.25 hinge variants are shown for 8 viruses. Medians are indicated and significance calculated using the Friedman test with Dunns correction for multiple comparisons are shown with *p<0.05 and **p<0.01. **(F)** Mean and geomean hinge variants IC50 as well as fold differences from IgG1 are shown grouped by hinge with red indicating increased and green decreased fold differences.

CAP256.25 IgG3 variants showed the greatest increase in neutralization potency over IgG1 for 3 viruses that all lacked a PNG motif at position 160 (Fig 5A and S2 Table). This glycan forms part of the epitope for this bNAb and is highly conserved [37]. Introduction of the PNG motif into these 3 viruses, CAP256_PI, CAP8.6F and CA146.H3.3, resulted in similar neutralization potencies for IgG1 and IgG3 (Fig 5B and S3 Table). However, deletion of the PNG motif in the CAP256_SU virus did not introduce preferential neutralization by IgG3 (S3 Table). Only one other virus in our panel, BG505, lacked N160 but did not show enhanced IgG3 neutralization, suggesting that the absence of this glycan alone is not sufficient for differential neutralization by IgG subclasses. Nevertheless, these data indicate that viral determinants also contribute to the increased potency of IgG3 variants.

### Increased hinge length of IgG3 confers both enhanced Fc effector function and neutralization potency

Others have suggested that enhanced neutralization potency and/or Fc effector function of IgG3 antibodies may be a consequence of increased hinge length and flexibility [44, 51, 52]. Given that binding to Fcγ receptors was only moderately improved for IgG3 variants we examined the effect of hinge length on the ability of a bNAb to modulate Fc function. CAP256.25 hinge switch variants were engineered and tested against BG505.SOSIP.664 for ADCP and ADCT activity. The IgG3*01 and IgG3*01m variants have hinge lengths of 62 aa while the IgG3*17 hinge is 47 aa and that of IgG1 is 16 aa (Fig 5C). Introduction of the 47 and 62 aa IgG3 hinges into IgG1 significantly enhanced both ADCP and ADCT activity, while inserting the shorter IgG1 hinge into IgG3 variants significantly reduced their activity (Fig 5D). We also tested these hinge variants for neutralization using eight viruses that had shown enhanced neutralization by IgG3 variants (CAP256_SU virus served as a control with no difference between subclasses) (S4 Table). Similar to ADCP and ADCT, insertion of longer IgG3 hinges into the CAP256.25 IgG1 variant led to significant increases in neutralization potency compared to IgG1 (Fig 5E and 5F). The introduction of the short IgG1 hinge into both the IgG3 bNAbs resulted in significant decreases in potency. Overall, these data indicate that enhancement of Fc effector function and neutralization by IgG3 variants of CAP256.25 is largely attributed to the naturally long hinge region of IgG3.

## Discussion

HIV antibody development for passive immunization has largely focused on neutralization activity, but the contribution of the constant region to protection is becoming increasingly clear [19]. The HIV-infected donor CAP256 provided a unique opportunity to explore the role of IgG3 since both neutralization breadth and Fc effector functions were shown to be mediated by this subclass in this individual. Using V2-specific antibodies previously isolated from donor CAP256, we showed that the IgG3 constant region improved both Fc effector function and neutralization potency. This effect was primarily the result of the increased hinge length of IgG3. These data highlight features of the constant region that could be exploited to enhance the functionality of antibodies being developed for passive immunity.

In this study, we show that IgG subclass had a significant impact on Fc effector function, with IgG3 variants of CAP256-VRC26 bNAbs better able to mediate ADCP and ADCT compared to IgG1. This observation confirms several studies showing HIV-specific mAbs expressed as IgG3 mediate higher levels of ADCP [44–46] (Chu, co-submission). ADCP is highly relevant to HIV prevention strategies since it can be induced by vaccination [53] and mucosal effector cells demonstrate potent HIV-specific ADCP [54]. IgG3 also showed enhanced ADCT, a newly-described HIV Fc-dependent function that results in rapid killing through removal of membrane fragments from target cells [42]. We have previously shown an association between plasma levels of IgG3 and ADCT in HIV-infected donors [55] and these new findings using isolated antibodies consolidate this link. Although improved ADCP and ADCT was mirrored by enhanced affinity for FcγRIIa/b, other factors also contributed to this enhanced functionality. One factor that did not impact the IgG3-mediated ADCP [44](Chu, co-submission) or ADCT was antibody’s affinity for antigen. Others have speculated that enhanced ADCP by IgG3 is due to the longer hinge length [44] which we show in this study and Chu, et al. confirms using HIV antibodies of different specificities, suggesting that this observation may be more broadly applicable (Chu, co-submission). In addition, increased hinge length of IgG3 contributed to enhanced binding to FcγRII [56], consistent with our observations of higher ADCP and ADCT activity. The ability of Fc receptors to cluster at the cell surface is a requirement for ADCP activity [57], where crosslinking of these receptors may be enhanced by multimers consisting of antibodies with extended hinges. Cumulatively, these data suggest that the mechanism for improved Fc effector function by IgG3 antibodies, in combination with improved FcγR affinity, is increased hinge length. This may provide enhanced flexibility leading to greater avidity effects such as the ability to engage multiple envelope spikes.

In addition to affecting Fc function, we show a role for IgG3 subclass in modulating antibody neutralization potency in the CAP256.25 and CAP256.29 bNAbs. While earlier studies have shown a similar effect for IgG3, these used weakly neutralizing HIV antibodies, F105 and F240 rather than bNAbs [58, 59]. Class switch variants using other isotypes such as IgA versions of bNAbs CH31, 2G12 and 2F5 have also shown improved affinity, neutralization and protection compared to IgG [60–62]. However, some MPER bNAbs are unaffected as subclass switch variants [63], suggesting the impact of the constant region may depend on the epitope targeted. The lack of any impact of class switch variation on neutralization was also seen for the CD4 binding site specific VRC01 and V3-glycan specific 447-52D, as described by Chu et al. (Chu, co-submission). This may be due to differences in binding stoichiometry between different antibodies, some of which may derive greater benefit from increased flexibility in the hinge than others [36, 64]. Regardless, these data provide substantial evidence that the constant regions of HIV-specific mAbs can influence their neutralization activity. This finding has implications for the selection of suitable antibody subclasses for passive immunization where increased potency translates into a lower therapeutic dose [65].

For some viruses, IgG3 CAP256 bNAb variants showed as much as 20-fold increase in potency compared to IgG1. These viruses lacked a PNG at position 160 in the V2 loop and were most potently neutralized by IgG3 variants of the CAP256-VRC26 lineage. This glycan may sterically hinder the angle at which the long-hinged IgG3 variants approach the trimer. The finding that interaction with viral epitopes is altered by isotype supports previous studies of an IgA variant of bNAb 2F5 that had a distinct paratope compared to the IgG variant [61]. We also confirmed that the longer hinge length of the IgG3 subclass variants, IgG3*01m and IgG3*17 mediated enhanced neutralization potency. This supports the possibility that the flexibility of IgG3 increases the accessibility of this antibody paratope, distinct from those bound by hinge variants of VRC01 and 447-52D which do not differ in neutralization potency (Chu, co-submission). In line with this finding, Scharf and colleagues showed that the hinge contributed to improved neutralization by comparing the Fab and F(ab')₂ (which includes the hinge) fractions of polyclonal IgG3 from HIV infected individuals [52]. Introduction of an IgG3 hinge was also recently used to significantly enhance the potency and protection of a HIV bispecific IgG1 bNAb [66]. The impact of increased hinge length may also depend on the density of the target antigen, as has been shown for the IgG3 which was more bactericidal than IgG1 when directed against a sparsely distributed meningococcal antigen [67]. Given that the trimer is also sparsely arranged on the surface of HIV [68], the IgG3 subclass may provide an advantage by improving binding and neutralization to trimeric proteins. This suggests that the hinge acts a bridge to enhance both Fab to Fab [34] and Fab to Fc flexibility which may simultaneously affect cross-linking of trimer and Fc receptor binding.

Several studies have examined the impact of serologically defined IgG allotypes on function, disease susceptibility and response to HIV vaccination, highlighting the relevance of genetic variation in the constant region [21, 69]. However, IgG allotypes encompass multiple alleles and do not fully describe allelic variation [30]. An example is N392K, a SNP that does not define any allotype but showed reduced ADCC function of IgG3*17. Here, we show that IgG3 allelic variation in both the CH3 and the hinge of IgG3 has functional relevance, which suggests that it may impact on the efficacy of passive immunization or individual responses to vaccination. The description of a novel IgG3 allele in this study shows the importance of sequencing constant regions of African populations, which are under-represented in genomic databases.

The variable and constant regions of antibodies are often treated as two structurally and functionally distinct components, despite growing evidence of co-operation between them [18, 70]. We recently showed that early and potent Fc effector polyfunctionality predicted the development of neutralizing activity, highlighting that these different functions of antibodies are intrinsically linked [55]. This study confirms that monoclonal antibodies with identical variable regions but different IgG subclasses and alleles can mediate enhanced Fc effector function and neutralization, modulated by the hinge length. This provides rationale for isolating antibodies as they naturally occur. IgG3 is not currently used for any therapeutic mAbs primarily because of short half-life and large number of alleles which may result in anti-allotypic effects. However, IgG3 variants such as IgG3*17 have a half-life equivalent to IgG1 and there is currently no evidence that allelic mismatch causes any clinical adverse effects in therapy, illustrating that IgG3 should be considered for passive immunization. In addition, this and the related study by Chu, et al.. (Chu co-submission) provide justification for the use of increased hinge length to improve antibody function. Ultimately, this study illustrates the strength of leveraging genetic variability to improve antibody function and shows that antibodies should be optimised based not only on their antigen binding characteristics but also on the intrinsic properties of their constant regions.

## Materials and methods

### Ethics statement

The CAPRISA 002 study was approved by the Biomedical Research Ethics Committee of the University of KwaZulu-Natal (M160791) and this specific study was approved by the Human Research Ethics Committee of University of the Witwatersrand (M150313). All participants were adults and provided written informed consent to have their stored samples used for future studies. All healthy subjects in this study were adults and provided written informed consent to donate both PBMCs and plasma and to have their samples stored for future use.

### Human subjects and sample preparation

For this study we used plasma from 23 HIV-positive women from the CAPRISA 002 acute HIV infection cohort study sampled at 6,12 and 36 months post-infection. IgG was isolated from plasma using Protein G in order to eliminate the cofounding impact of cytokines or plasma proteins on cell-based assays, quantified by a Nanodrop spectrophotometer (Pierce Biotechnology, Rockford, IL) and confirmed by IgG ELISA.

Additionally, whole peripheral blood mononuclear cells (PBMCs) from a healthy male donor were used as effector cells for the ADCC assays. The FcγRIIIa receptor was genotyped as being homozygous for valine at position 158 by the TaqMan SNP genotyping assay (rs396991) (Applied Biosystems, Foster City, CA) to ensure high levels of lysis. Similarly, plasma from a healthy female donor was used as a source of complement.

### Cell lines

THP-1 cells obtained from the AIDS Reagent Program (Division of AIDS, NIAID, NIH contributed by Dr. Li Wu and Vineet N. KewalRamani) were used for both the ADCP and ADCT assays. Cells were cultured at 37°C, 5% CO2 in RPMI containing 10% heat-inactivated fetal bovine serum (Gibco, Gaithersburg, MD), 1% Penicillin Streptomycin (Gibco, Gaithersburg, MD) and 2-mercaptoethanol to a final concentration of 0.05 mM. CEM-NK_R_.CCR5, a CEM-natural killer resistant T lymphoblast cell line transduced with CCR5 served as targets in the ADCC, ADCT and ADCD assays. These were obtained from the AIDS Reagent Program (Division of AIDS, NIAID, NIH developed by Dr Alexander Trkola) and were cultured at 37°C, 5% CO2 in RPMI containing 10% heat-inactivated fetal bovine serum (Gibco, Gaithersburg, MD) and 1% Penicillin Streptomycin (Gibco, Gaithersburg, MD). TZM-bl cells, previously designated JC53-bl (clone 13) cells, are a HeLa cell line expressing high levels of CD4 and CCR5 and transduced with a luciferase gene under the control of the HIV promoter. These were obtained from the AIDS Reagent Program (Division of AIDS, NIAID, NIH developed by Dr. John C. Kappes, and Dr. Xiaoyun Wu). These cells TZM-bl cells transduced with FcγRI, FcγRIIa, FcγRIIb and FcγRIIIa were a gift from Dr. David Montefiori (Duke University, Durham, NC) and were prepared as described previously [50]. All of these cell lines were used for neutralization assays. HEK293T cells were obtained from Dr. George Shaw (University of Alabama, Birmingham, AL) and were used for pseudovirus expression. These adherent cell lines were cultured at 37°C, 5% CO2, in DMEM containing 10% heat-inactivated fetal bovine serum (Gibco BRL Life Technologies) and supplemented with 50 μg/ml gentamicin (Sigma). Cells were disrupted at confluence with 0.25% trypsin in 1 mM EDTA (Sigma) every 48–72 hours. HEK293F suspension cells were cultured in 293Freestyle media (Gibco BRL Life Technologies) and grown in a shaking incubator at 37°C, 5% CO2, 70% humidity at 125rpm.

### Proteins and peptides

Plasmids encoding histidine-tagged recombinant gp120 from ConC and gp120 from the CAP45.G3 envelope sequences were transfected using polyethylenimine 25 kDa (Polysciences Inc, Warrington, PA) into HEK293T cells. Recombinant proteins were expressed and purified as previously described [71]. BG505.SOSIP.664 gp140 trimer was produced in HEK293F suspension cells and purified by size exclusion chromatography (SEC) [64]. Prior to use, the trimer was subject to quality control by ELISA binding of CAP256.25 and PGT151 and the lack of binding to F105. The C.ZA.1197MB strains of gp41, p24 and gp140 were purchased from Immune Tech (Lexington, New York), CAP88.B5 V3 peptide, CAP248 MPER peptide and MPR.03 were purchased from Peptide 2.0 (Chantilly, Virginia). V1V2 scaffolded proteins and peptides including cyclic CAP45.G3, cyclic ConC, gp70V1V2 p2863, gp70V1V2 caseA2, AE 244a and Fda6-CAP45 were expressed in HEK293S cells (ATCC CRL-3022 N-acetylglucosaminyltransferase I-deleted), grown in a shaking incubator at 37°C, 5% CO2, 70% humidity at 125rpm. Cultures were harvested after seven days and purified by sequential Ni-NTA and SEC.

### Customised multiplex IgG subclass assay

A customised multiplex assay was used as previously described [38]. Briefly, multiplex microplex carboxylated beads (Luminex, Madison, WI) were coupled to14 HIV specific antigens including gp120 (ConC and CAP45.G3), V2 peptides and scaffolds (cyclic CAP45.G3, cyclic ConC, gp70V1V2 p2863, gp70V1V2 caseA2, AE 244a and Fda6-CAP45), V3 peptide (CAP88.B5), gp41 (CAP248 MPER peptide, MPR.03 peptide and C.ZA.1197MB gp41), gp140 (C.ZA.1197MB) and p24 (C.ZA.1197MB). The bead preparation (50μl of 100 microspheres/μl) was incubated with purified IgG overnight (100μg/μl) at 4°C. Levels of bulk IgG and IgG1-IgG4 subclasses were detected by PE-conjugated detection agents (Southern Biotech, Birmingham, AL) by a Bio-Plex200. The mean of PBS only samples added to 3 times their standard deviation was subtracted from all samples as background.

### IgG3-specific ELISA

Total IgG3 was measured by coating a high binding ELISA 96-well plate with an anti-human IgG mAb (Sigma-Aldrich, St Louis, MO) overnight at 4°C. Similarly, to measure IgG3 specific responses against other diseases, ELISA plates were coated with cytomegalovirus glycoprotein B (ab43040), measles virus Priorix, Schwarz strain nucleocapsid protein (ab74559) or Influenza A virus hemagglutinin H1 protein (ab69741) (Abcam, Cambridge, MA). Plates were blocked with 5% milk/ 0.05% Tween-20 in PBS for 1 hour at 37°C and plasma or isolated IgG was diluted in blocking buffer and incubated for a further hour. Following washes in 0.05% Tween 20 in PBS, IgG3 was detected with anti-human IgG3 HRP (Southern Biotech, Birmingham, AL) and following a further hour incubation TMB substrate was added and the reaction stopped with 1M H_2_S0_4_. To normalize for IgG levels in individuals, total IgG levels for all samples was measured as above with the exception of detecting with anti-human IgG-HRP (Sigma-Aldrich, St Louis, MO).

### IgG3 depletions

IgG3 capture beads were made as previously described [13]. M-270 Streptavidin Dynabeads (10mg) (Invitrogen) were washed 3 times in 500μl PBS. Following resuspension in 500μl PBS, beads were incubated with biotin conjugated anti-human IgG3 (Southern Biotech, Birmingham, AL) at room temperature rotating for 1 hour. Beads were washed 3 times in 0.1% PBS/BSA. Beads were reconstituted at 10mg/ml and 100μl was incubated with end over end rotation for 24 hours with 120μl (1mg/ml) of polyclonal IgG. Successful depletion of IgG3 from IgG isolations was confirmed by IgG3 ELISA and the total IgG concentrations also adjusted for depletion for input to downstream assays.

### Antibody-dependent cellular phagocytosis assay (ADCP)

The THP-1 phagocytosis assay was performed as in [41] using 1μM neutravidin beads (Molecular Probes Inc, Eugene, OR) coated with BG505.SOSIP.664 gp140 trimer. Polyclonal IgG samples were titrated and tested at a final concentration of 100μg/ml. Additionally monoclonal antibodies were tested starting at 100μg/ml with 5-fold dilutions. Phagocytic scores were calculated as the geometric mean fluorescent intensity (MFI) of the beads that have been taken up multiplied by the percentage bead uptake. This, as well as all other flow cytometry work was completed on a FACSAria II (BD Biosciences, Franklin Lakes, New Jersey). Pooled IgG from HIV-positive donors from the NIH AIDS Reagent programme (HIVIG) was used in all assays to normalize for plate to plate variation while samples from 10 Clade C-infected individuals was used as a positive control for all assays. Palivizumab (MedImmune, LLC; Gaithersburg, MD) was used as negative control. In addition, F105 and A32 were used to determine the structural integrity of BG505.SOSIP.664 trimer.

### Antibody-dependent cellular cytotoxicity (ADCC)

ADCC activity was detected by the ADCC-GranToxiLux (GTL) assay using antigen-coated cells as described in [43]. Whole PBMCs from a healthy donor were used as effector cells. Target CEM-NKR.CCR5 cells were coated with BG505.SOSIP.664 at 10μg/ml respectively. Optimal coating concentration was determined by titration of the antigen and measuring residual levels of unbound CD4 with anti-CD4 FITC (SK3 clone, BD Biosciences). The results, analysed in FlowJo (FlowJo LLC, Ashland, Oregon) are expressed as % Granzyme B (GzB) activity, defined as the percentage of cells positive for proteolytically active GzB out of the total viable target cell population. The final results are expressed after subtracting the background represented by the % GzB activity observed in wells containing effector and target cell populations in the absence of IgG.

### Antibody-dependent complement deposition (ADCD)

ADCD was determined by the deposition of the complement component C3b on the surface of CEM-NK_R_.CCR5 cells as described in [10]. Target cells were pulsed with 10μg BG505.SOSIP.664 gp140 trimer in 100μl of R10 media (10% FBS 1% Pen/Strep RPMI, Gibco, Gaithersburg, MD) for 1 hour at room temperature and incubated with 100μg/ml of IgG preparation. HIV-negative plasma was used as a source of complement and diluted 1 in 10 in 0.1% gelatin/veronal buffer (Sigma-Aldrich, St Louis, MO) and 150μl added and incubated for 20 minutes at 37°C. The cells were then washed in 15mM EDTA in PBS and C3b was detected by flow cytometry using an anti-human/mouse complement component C3/C3b/iC3b mAb (Cedarlane, Burlington, Canada). Unpulsed cells were used as background controls and HIV-negative plasma was heat-inactivated at 56°C to remove complement as a negative control. The ADCD score was defined as geometric MFI multiplied by % cells positive for C3b deposition.

### Antibody-dependent cellular trogocytosis (ADCT)

CEM-NK_R_.CCR5 cells were pulsed with BG505.SOSIP.664 trimer (10μg/ml) for 75 minutes at room temperature [42]. Optimal coating concentrations were determined as described above. Cells were stained with PKH26 dye (Paul Karl Horan 26 dye) as per instructions from the manufacturer (Sigma-Aldrich, St Louis, MO) and resuspended at 2 million cells/ml. IgG at a final concentration of 100μg/ml was added to the cells and incubated for 30 minutes at 37°C. THP-1 cells were stained with intracellular CFSE (carboxyfluorescein succinimidyl ester) and 150μl containing 6.7 x 10^5^ cells/ml was added to the plate and incubated for a further hour at 37°C. Cells were then washed with 15mM EDTA in PBS. Flow cytometry was used to distinguish PKH26+ CFSE+ THP-1 cells (i.e. the uninfected monocytes that have received membrane fragments from the coated cells) and are represented as a proportion of total THP-1 cells. Doublets were excluded from the analysis by singlet gating. Cells were gated on stained CEM and THP-1 cells incubated in the absence of IgG to ensure that we did not measure antibody-independent trogocytosis. Uncoated PKH26 stained CEM cells were also incubated with THP-1 cells in the presence of HIV-specific IgG in order to ensure that the responses seen were HIV-specific.

### Pseudovirus production and site-directed mutagenesis

Pseudovirus plasmids expressing the HIV Env of interest were co-transfected with pSG3DEnv backbone-expressing plasmids (obtained from the NIH AIDS Research and Reference Reagent Program, Division of AIDS, NIAID, NIH) into 293T cells using PEI-MAX 40,000 (Polysciences). Cultures were incubated for 48 hours at 37°C, then filtered through 0.45 μm and frozen in DMEM/ 20% FBS to yield Env-pseudotyped viruses capable of a single round of infection only. Mutant envelope genes were generated with the QuikChange Lightning Kit (Stratagene), confirmed by DNA sequencing, and transfected as above.

### Neutralization assays

Neutralization assays were performed in TZM-bl cells as previously described (Montefiori, 2005). Neutralization is measured as a reduction in relative light units after a single round of pseudovirus infection in the presence of the monoclonal antibody or IgG sample of interest. IgG samples were serially diluted 1:3 while bNAbs were serially diluted 1:5 and the IC_50_ calculated as the dilution at which the infection was reduced by 50%. Variation in this assay was confirmed to be less than 3-fold. All subclass switch variants were run blinded and head-to-head on the same plate to limit intra-experimental variation. Neutralizations carried out in Fc receptor-transduced TZM-bl cells were performed exactly as above.

### Sequencing of germline CAP256 constant regions

Genomic DNA was extracted from PBMC using a Qiagen Total Allprep DNA RNA kit. DNA was quantified and 20ng used for each amplification reaction with HF Platinum Taq polymerase. A total of 12 PCR primers in the intron regions were used to amplify the CH1-3 regions of IgG1-3. The CH1 and CH2-CH3 amplicons were Sanger sequenced and the presence of SNPs noted with nucleic acid codes used to represent ambiguous bases.

### Sequencing of the constant region of isolated mAbs

MAbs were isolated from CAP256 as previously described [37]. The original cDNA of sorted memory B cells from which antibodies 29, 30 and 33 were isolated was amplified with IGHV3 forward and IgG constant reverse primers. Antibody 29 was amplified with primers VH3-leader B and CH3 rev 2 for 50 cycles using Qiagen HotStarTaq Plus DNA polymerase. Five ul of the first round PCR reaction was then amplified with primers 5’L-VH3 and CH3 rev 3 for a further 50 cycles. Antibodies 30 and 33 were amplified with the same forward primers but reverse primers positioned at the end of CH1 –CH1 rev 1 for the first round and CH1 rev 2 for the second.

VH3-leader B—TAAGAGGTGTCCAGTGT [72]

5’L-VH3 –AAGGTGTCCAGTGTGARGTGCAG [73]

CH3 rev 2—GGT TGT GCA GAG CCT CAT GCA TCA C

CH3 rev 3—CCT CAT GCA TCA CGG AGC ATG AGA AG

CH1 rev 1—CTC TTG TCC ACC TTG GTG TTG CTG

CH1 rev 2—CTG GGC TTG TGA TTC ACG TTG CAG G

### Cloning and mAb expression

The heavy and light chain variable regions of mAbs of interest were cloned into both an IgG1 and IgG3*01 expression vector (received from Dr Bart Haynes, Duke University, Durham, NC). The plasmid was subjected to site-directed mutagenesis to obtain IgG3*01m. Hinge mutant and IgG3*17 constructs were ordered from GenScript (Piscataway, NJ). For antibody expression, plasmids separately encoding heavy and light chain genes were co-transfected into 293F cells with PEI-MAX 40,000 (Polysciences). Cells were cultured for six days in 293Freestyle media at 37°C, 10% CO2, then harvested supernatants were filtered and purified using Protein G (Thermoscientific). All variants were quantified by nanodrop using sequence specific extinction coefficients as determined by ProtParam (ExPASy) and confirmed by ELISA.

### BG505.SOSIP.664 ELISA

Avitagged BG505.SOSIP.664 trimer was biotinylated using BirA ligase as described elsewhere [37]. Biotinylated trimer was coated on to streptavidin ELISA plates (Thermofisher) at 4μg/ml in PBS and incubated for 1 hour at room temperature. Following PBS washes, the plates were blocked for 30 minutes in 5% milk/PBS and washed in PBS. Fifty μl of CAP256.29 and CAP256.25 variants as well as negative controls 447-52D and F105 (starting at 20μg/ml) were incubated for 1 hour at room temperature, followed by PBS washes. Secondary antibody, goat anti-human F(ab’)2 HRP was incubated in the plate for 1 hour at room temperature, the plate washed three times with PBS and 100μl TMB added to each well. The reaction was stopped with 1M H_2_S0_4_ and read at 450nm.

### Kinetic measurements

Surface plasmon resonance (SPR) was used to measure equilibrium binding affinities (as previously described [45]. A Continuous Flow Microspotter (CFM) (Carterra, Salt Lake City, UT) was used to print up to 96 individual regions of interest (ROI) on a single gold prism surface functionalized with carboxymethyldextran substrate (CMD200M, Xantec Bioanalytics, Dusseldorf, DE). CFM fluid paths were primed with 25 mM sodium acetate pH 5.0 + 0.01% Tween 20 before activation. The substrate of each ROI was activated for 5–7 min with 100 μL of 1.2 mM N-hydroxysulfosuccinimide (NHS) (Pierce) and 0.3 mM 1-ethyl-3-[3 dimethlyaminopropyl]carbodiimide-HCl (EDC) (Pierce) in 10 mM 2-(N-morpholino)ethanesulfonic acid (MES) pH 5.0 under flow at 45 μL/min. Antibodies were prepared at 200 nM in 25 mM sodium acetate pH 5.0 and printed on the activated substrate. The image-based array reader (MX96, IBIS Technologies, Enschede, NL) was primed with 25 mM sodium acetate pH 5.0 + 0.01% Tween 20 and the prism loaded and quenched with 120 μL of 1 M ethanolamine (Sigma Aldrich), before priming, conditioning, and analyte injections. FcγRs were diluted in running buffer (PBS + + 0.05% Tween 20) and injected over an 8-part, 3-fold serial dilution series, generally starting between 10 and 50 μM, and consisting of the following steps: 0.5 min baseline, 5 min association, 5 min dissociation, 0.5 min baseline. Each series of FcγR injections was followed by duplicate blank injections to ensure full dissociation of analyte. ROI signal was double referenced using signal from blank injections and signal from uncoupled interspots to account for nonspecific binding. A genetically aglycosylated human IgG1 (N297Q) and the LALA variant were used as negative controls as well as IgG from HIV-positive individuals as positive controls. Experiments were repeated 2 to 3 times with different conjugation densities and print pH conditions. Data from 2 separate experiments, with multiple print spots for each IgG sample, are presented.

### Quantification and statistical analysis

Analysis of all flow cytometry based experiments was done using FlowJo (FlowJo LLC, Ashland, OR). SPR data was processed in Scrubber 2 (Biologic Software Ltd, Canberra, AU) by kinetic analysis applying global analysis to determine the equilibrium dissociation constant K_D_. Sequencing results were analyzed with Sequencher 5.4.1. Fc polyfunctionality Z-scores were calculated by standardising each Fc effector function (where the mean of the function is subtracted from the individual value and divided by the standard deviation of the mean) and then adding all the Z-scores for each function per individual. All statistical analysis was performed in GraphPad Prism 6 (GraphPad Software, Inc, La Jolla, CA). All comparisons between groups were done with non-parametric tests including Mann-Whitney U tests (for two unmatched groups), Wilcoxon matched pairs signed rank test (for two matched groups) and Kruskal-Wallis tests with Tukey’s correction for multiple comparison or Friedman’s test for matched groups with Dunn’s correction. All confidence intervals were set to 95%. All correlations reported are non-parametric Spearman’s correlations and all statistical analysis was done with two-sided testing with using an alpha level of 0.05.

## Supporting information

S1 Fig IgG3 depletions results in decreased neutralization by CAP256 plasma IgG. The table indicates IC_50_ (μg/ml) of undepleted and IgG3-depleted CAP256 plasma IgG at 36 months p.i. against 8 viruses with potent neutralization indicated in red and knockout (KO) of neutralization indicated in blue. Significant fold reduction in neutralization is indicated in bold with representative neutralization (inhibition) curves for undepleted (black) and IgG3 depleted (red) CAP256 IgG against all viruses tested. Experiments are representative of three individual repeats.
(PDF)

S2 Fig *IGHG3* allele sequences from germline DNA and isolated antibodies from donor CAP256. CH1-CH3 sequence of germline *IGHG3* from donor CAP256 is shown along with constant regions of isolated antibodies and two of the most closely related *IGHG3* alleles. Amino acids are numbered according to the Eu system with the SNPs that are unique to the respective alleles are indicated. Colours correspond to IGHG3*01 (blue), IGHG3*01m (red) and IGHG3*17 (green).
(PDF)

S3 Fig Novel *IGHG3* allele identified in donor CAP256. All 29 *IGHG3* constant region alleles in the ImMunoGeneTics (IMGT) database are shown along with the novel IGHG3*01m allele identified in donor CAP256 as indicated in red. CH1, hinge, CH2 and CH3 regions are indicated. SNPs used to define IgG3 allotypes are highlighted in orange with potential N linked glycosylation sites shown in grey and positions are indicated using Eu numbering.
(PDF)

S4 Fig Fc effector functions of IgG1 and IgG3 CAP256 variants. Titrations of CAP256.29 and CAP256.25 IgG1 (black), IgG3*01 (blue), IgG3*01m (red) and IgG3*17 (green) variants and Palivizumab (negative control) for ADCP, ADCT, ADCC and ADCD activity against BG505.SOSIP.664 trimer are shown. Mean and standard deviation of 3 independent experiments are represented.
(PDF)

S5 Fig Representative SPR response curves and 1:1 stoichiometry kinetic model fits. **(A)** CAP256.25 mAb constant region variants were directly printed onto the SPR chip and analyzed for binding to FcγRIIa-R131. Raw curves (black) and kinetic fits (red) are shown for IgG1, IgG3*01m, IgG3*01 and IgG3*17, an aglycosylated Fc variant produced by N297Q point mutation and the Fc-engineered LALA mutant. **(B)** Standard deviations of dissociation equilibrium constants (K_D_ in μM) determined by SPR for all variants of CAP256.29 and CAP256.25 binding to 5 different Fc receptors. CAP256 polyclonal IgG was a positive control and VRC01 N297Q was a negative control. Data are representative of 2 independent experiments.
(PDF)

S6 Fig CAP256.25 IgG3*17 K392N significantly increases ADCC and binding to FcγRIIIa receptors. Position Lys-392 CAP256.25 IgG3*17 was mutated to Arg-392 and both were tested for **(A)** ADCP, ADCT, ADCD and ADCC as well as **(B)** binding by SPR to FcγRIIa (H131/R131), FcγRIIb and FcγRIIIa (F158/V158). Significance between wild type and mutant were calculated by the Wilcoxon signed-rank test where *<p<0.05; **p<0.01. Bar represent means with error bars indicating standard deviations of 2 or 3 independent experiments.
(PDF)

S1 Table IC_50_ and fold differences of bNAbs (A) CAP256.25 and (B) CAP256.29 IgG1 and IgG3 allelic variants.
(XLSX)

S2 Table Sequence alignments of viruses and corresponding fold differences of IgG3 variant IC_50_ compared to CAP256.25 IgG1. (XLSX)

S3 Table PNG160 introduction into viruses abolishes increased IgG3 neutralization potency over IgG1. (XLSX)

S4 Table Introduction of increased hinge lengths of IgG3 mediates enhanced neutralization potency. (XLSX)
