# Supplementary material for: IgG3 enhances neutralization potency and Fc effector function of an HIV V2-specific broadly neutralizing antibody
Source: PLoS Pathog. 2019 Dec 16;15(12):e1008064. doi: 10.1371/journal.ppat.1008064 (PMC6936867; doi:10.1371/journal.ppat.1008064)

CAP256 36 months p.i

	Undepleted (IC50 μg/ml)	IgG3 depleted (IC50 μg/ml)	Fold reduction
CAP256 _SU	1.1	2.4	**2.2**
ZM53.12	1.8	8.0	**4.5**
ConC	1.8	10.7	**5.9**
CAP45.G3	2.9	>25	**KO**
CAP210.E8	3.7	17.0	**4.6**
BG505 N332+	13.9	>25	**KO**
Q23.17	14.1	>25	**KO**
MLV	>25	>25	-

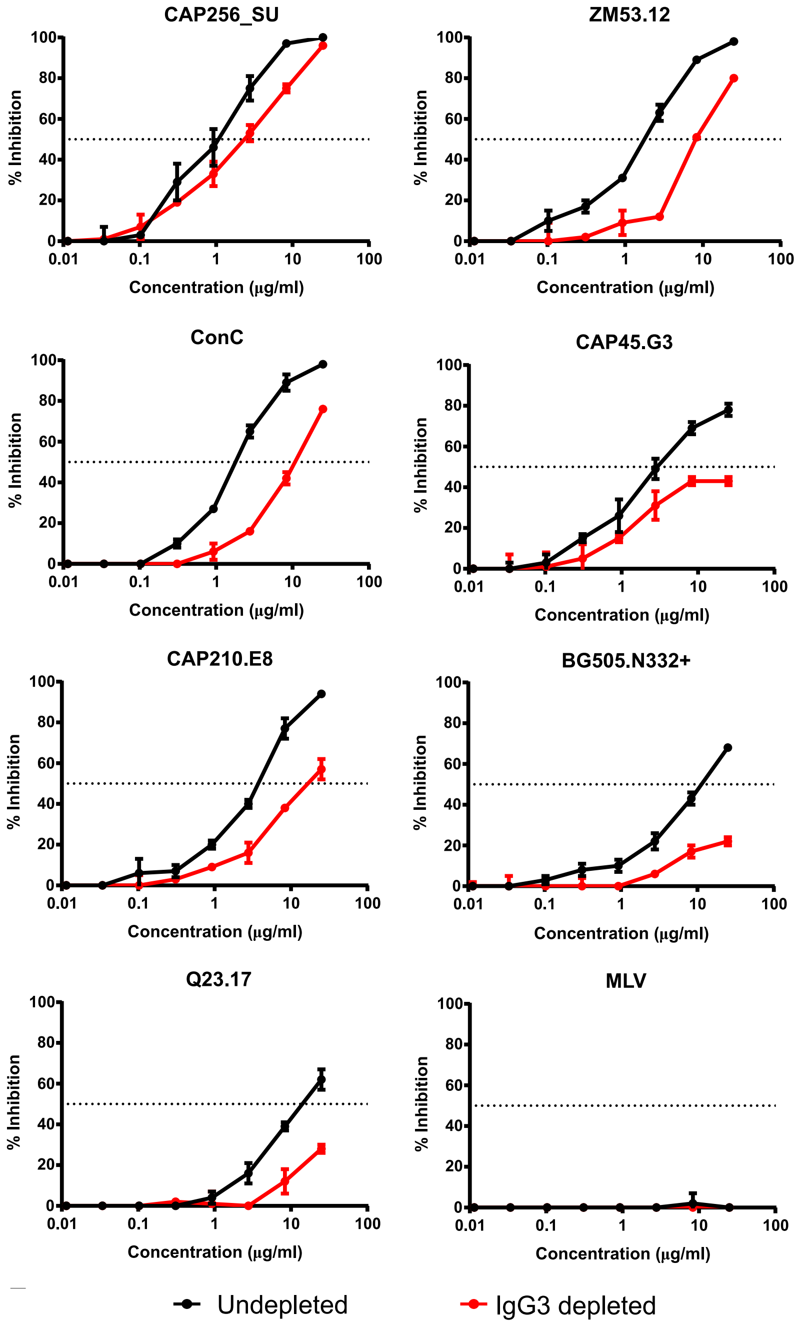

Supplement: S1 Fig — The table indicates IC50 (μg/ml) of undepleted and IgG3-depleted CAP256 plasma IgG at 36 months p.i. against 8 viruses with potent neutralization indicated in red and knockout (KO) of neutralization indicated in blue. Significant fold reduction in neutralization is indicated in bold with representative neutralization (inhibition) curves for undepleted (black) and IgG3 depleted (red) CAP256 IgG against all viruses tested. Experiments are representative of three individual repeats. (PDF) [file ppat.1008064.s001.pdf]
