# Supplementary material for: IgG3 enhances neutralization potency and Fc effector function of an HIV V2-specific broadly neutralizing antibody
Source: PLoS Pathog. 2019 Dec 16;15(12):e1008064. doi: 10.1371/journal.ppat.1008064 (PMC6936867; doi:10.1371/journal.ppat.1008064)
Supplement: S2 Fig — CH1-CH3 sequence of germline IGHG3 from donor CAP256 is shown along with constant regions of isolated antibodies and two of the most closely related IGHG3 alleles. Amino acids are numbered according to the Eu system with the SNPs that are unique to the respective alleles are indicated. Colours correspond to IGHG3*01 (blue), IGHG3*01m (red) and IGHG3*17 (green). (PDF) [file ppat.1008064.s002.pdf]

CH1

		192	193	
**CAP256 germline IgG3 SNPs**	ASTKGPSVFPLAPCSRSTSGGTAALGCLVKDYFPEPVTVSWNSGALTSGVHTFPAVLQSSGLYSLSSVVTVPSS	N/S	F/L	GTQTYTCNVNHKPSNTKVDKRV
IGHG3*01	....	S	L	....
CAP256.29 bNAb	....	S	L	....
CAP256.30 bNAb	....	S	L	....
CAP256.33 bNAb	....	S	L	....
IGHG3*17	....	N	F	....
1A2	....	N	F	....
1B10	....	N	F	....
1G2	....	N	F	....
1H	....	N	F	....

CH2

**CAP256 germline IgG3 SNPs**	APELLGGPSVFLFPPKPKDTLMISRTPEVTCVVVDVSHEDPEVQFKWYVDGVEVHNAKTKPREEQYNSTFRVVSVLTVLHQDWLNGKEYKCKVSNKALPAPIEKTISKTKGQ
IGHG3*01	....
CAP256.29 bNAb	....
IGHG3*17	....
1A2	....
1B10	....
1G2	....
1H	....

CH3

		379		392		397		419	
**CAP256 germline IgG3 SNPs**	PREPQVYTLPPSREEMTKNQVSLTCLVKGFYPSDIA	M/V	EWESSGQPENNY	K/N	TTPP	V/M	LDSDGSFFLYSKLTVDKSRWQ	Q/E	GN
IGHG3*01	....	V	....	N	....	M	....	Q	..
CAP256.29 bNAb	....	V	....	N	....	M	....	E	..
IGHG3*17	....	M	....	K	....	V	....	Q	..
1A2	....	M	....	K	....	V	....	Q	..
1B10	....	M	....	K	....	V	....	Q	..
1G2	....	M	....	K	....	V	....	Q	..
1H	....	M	....	K	....	V	....	Q	..

		435	436	
**CAP256 germline IgG3 SNPs**	IFSCSVMHEALHN	H/R	Y/F	TQKSLSLSPGK
IGHG3*01	....	R	F	....
IGHG3*17	....	H	Y	....
