# Supplementary figures and images for: IgG3 enhances neutralization potency and Fc effector function of an HIV V2-specific broadly neutralizing antibody
Source: PLoS Pathog. 2019 Dec 16;15(12):e1008064. doi: 10.1371/journal.ppat.1008064 (PMC6936867; doi:10.1371/journal.ppat.1008064)

CAP256.29

CAP256.25

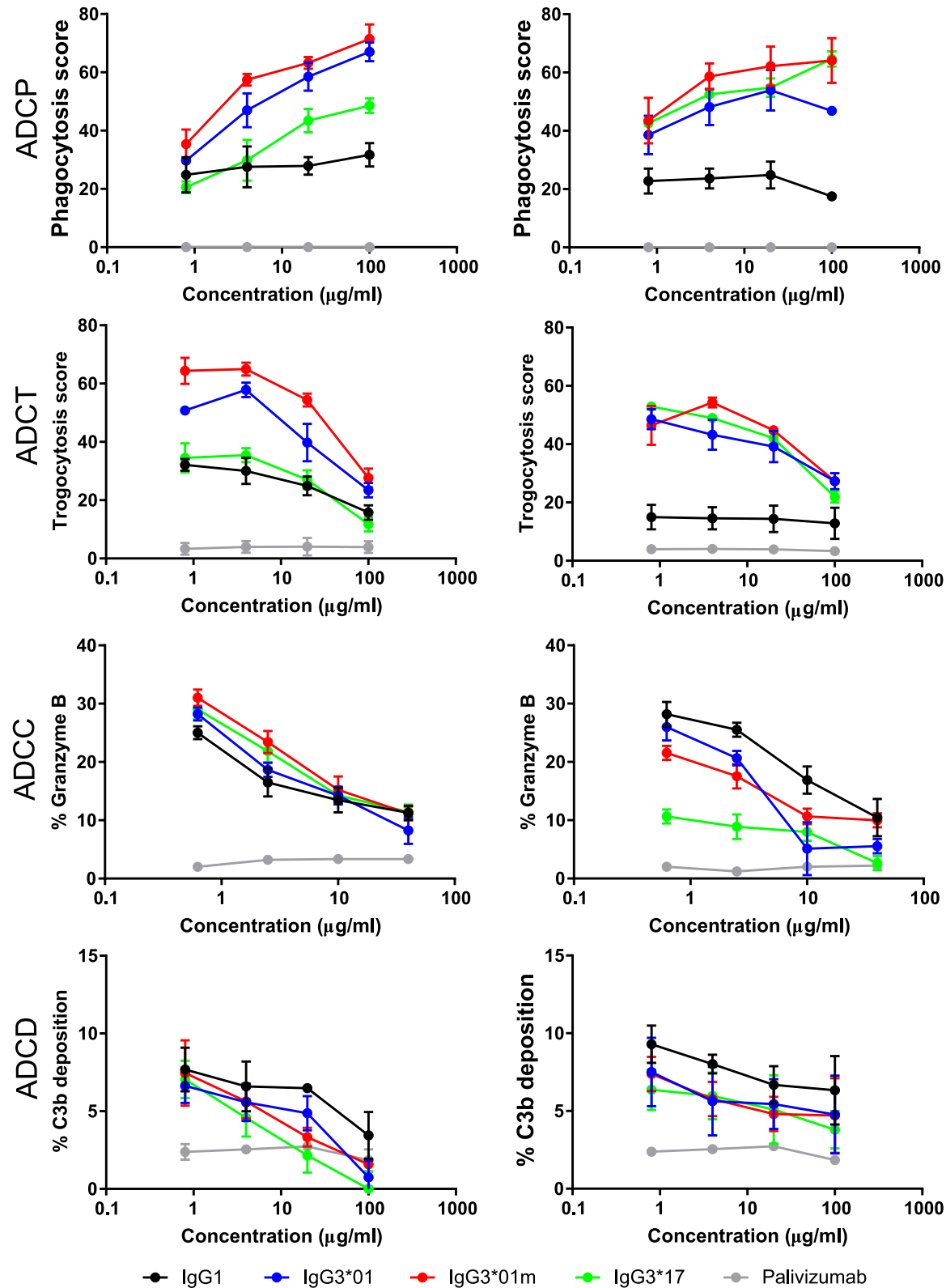

Supplement: S4 Fig — Titrations of CAP256.29 and CAP256.25 IgG1 (black), IgG3*01 (blue), IgG3*01m (red) and IgG3*17 (green) variants and Palivizumab (negative control) for ADCP, ADCT, ADCC and ADCD activity against BG505.SOSIP.664 trimer are shown. Mean and standard deviation of 3 independent experiments are represented. (PDF) [file ppat.1008064.s004.pdf]

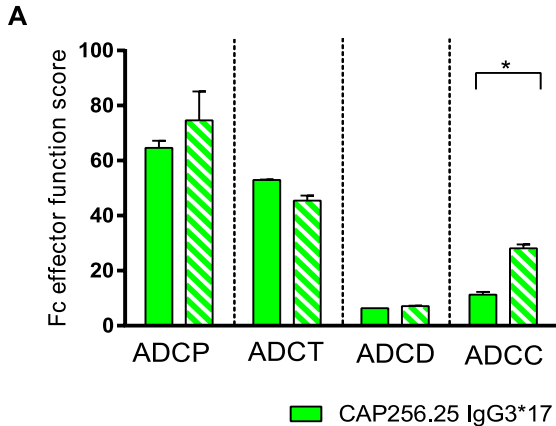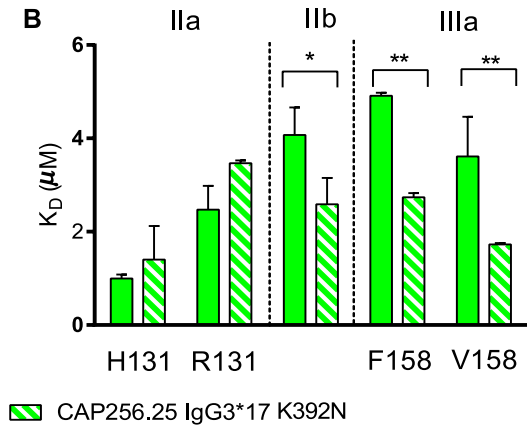

Supplement: S6 Fig — Position Lys-392 CAP256.25 IgG3*17 was mutated to Arg-392 and both were tested for (A) ADCP, ADCT, ADCD and ADCC as well as (B) binding by SPR to FcγRIIa (H131/R131), FcγRIIb and FcγRIIIa (F158/V158). Significance between wild type and mutant were calculated by the Wilcoxon signed-rank test where *<p<0.05; **p<0.01. Bar represent means with error bars indicating standard deviations of 2 or 3 independent experiments. (PDF) [file ppat.1008064.s006.pdf]
