# Supplementary material for: IgG3 enhances neutralization potency and Fc effector function of an HIV V2-specific broadly neutralizing antibody
Source: PLoS Pathog. 2019 Dec 16;15(12):e1008064. doi: 10.1371/journal.ppat.1008064 (PMC6936867; doi:10.1371/journal.ppat.1008064)
Supplement: S5 Fig — (A) CAP256.25 mAb constant region variants were directly printed onto the SPR chip and analyzed for binding to FcγRIIa-R131. Raw curves (black) and kinetic fits (red) are shown for IgG1, IgG3*01m, IgG3*01 and IgG3*17, an aglycosylated Fc variant produced by N297Q point mutation and the Fc-engineered LALA mutant. (B) Standard deviations of dissociation equilibrium constants (KD in μM) determined by SPR for all variants of CAP256.29 and CAP256.25 binding to 5 different Fc receptors. CAP256 polyclonal IgG was a positive control and VRC01 N297Q was a negative control. Data are representative of 2 independent experiments. (PDF) [file ppat.1008064.s005.pdf]

A

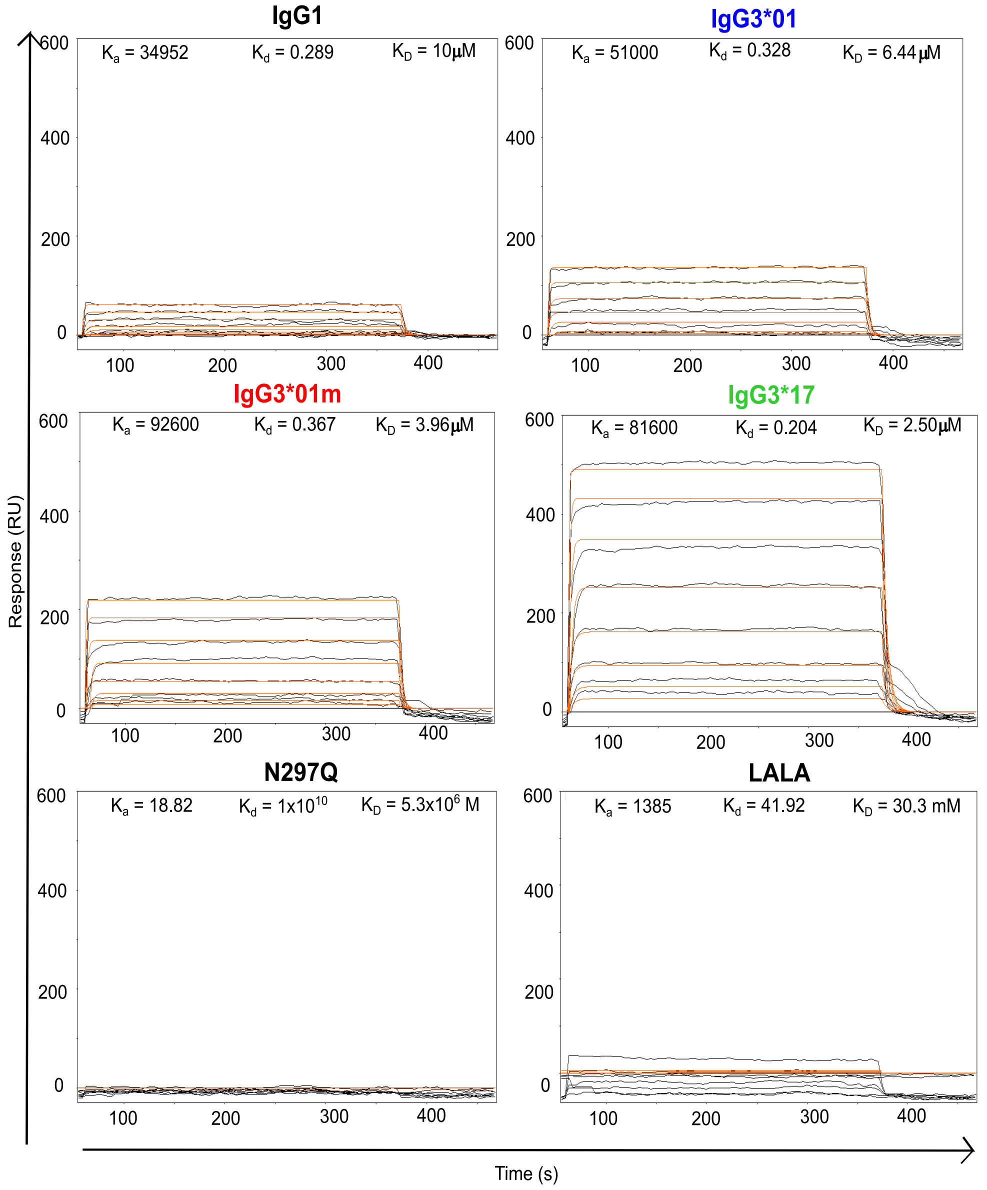

B

		CAP256.29 K_D (μM)				Controls K_D (μM)		CAP256.25 K_D (μM)			
**Receptor**	**Function**	**IgG1**	IgG3*01	IgG3*01m	IgG3*17	N297Q	CAP256 pIgG	**IgG1**	IgG3*01	IgG3*01m	IgG3*17
FcγRIIa H131	ADCT/ADCP	5.5 ± 0.8	1.3 ± 0.1	1.4 ± 0.1	1.3 ± 0.1	-	5.8 ± 0.8	5.6 ± 0.8	1.0 ± 0.1	1.0 ± 0.1	1.0 ± 0.1
FcγRIIa R131	ADCT/ADCP	5.2 ± 0.8	3.0 ± 0.2	3.2 ± 0.1	2.9 ± 0.3	-	4.6 ± 0.6	5.7 ± 0.2	3.9 ± 0.2	2.8 ± 0.1	2.0 ± 0.1
FcγRIIb	ADCT/ADCP	5.4 ± 0.3	3.1 ± 0.5	3.4 ± 0.5	4.1 ± 0.6	-	5.3 ± 0.6	6.6 ± 0.8	4.6 ± 0.4	4.8 ± 0.5	4.5 ± 0.4
FcγRIIIa F158	ADCC	4.5 ± 0.5	4.4 ± 0.7	3.9 ± 0.3	4.5 ± 0.7	-	4.5 ± 0.6	3.9 ± 0.5	3.1 ± 0.1	3.0 ± 0.1	4.9 ± 0.1
FcγRIIIa V158	ADCC	3.6 ± 0.5	2.6 ± 0.1	2.7 ± 0.2	2.4 ± 0.3	-	2.2 ± 0.3	2.4 ± 0.3	2.2 ± 0.2	2.1 ± 0.3	4.1 ± 0.5
