# Supplementary material for: IgG3 enhances neutralization potency and Fc effector function of an HIV V2-specific broadly neutralizing antibody
Source: PLoS Pathog. 2019 Dec 16;15(12):e1008064. doi: 10.1371/journal.ppat.1008064 (PMC6936867; doi:10.1371/journal.ppat.1008064)
Supplement: S3 Fig — All 29 IGHG3 constant region alleles in the ImMunoGeneTics (IMGT) database are shown along with the novel IGHG3*01m allele identified in donor CAP256 as indicated in red. CH1, hinge, CH2 and CH3 regions are indicated. SNPs used to define IgG3 allotypes are highlighted in orange with potential N linked glycosylation sites shown in grey and positions are indicated using Eu numbering. (PDF) [file ppat.1008064.s003.pdf]

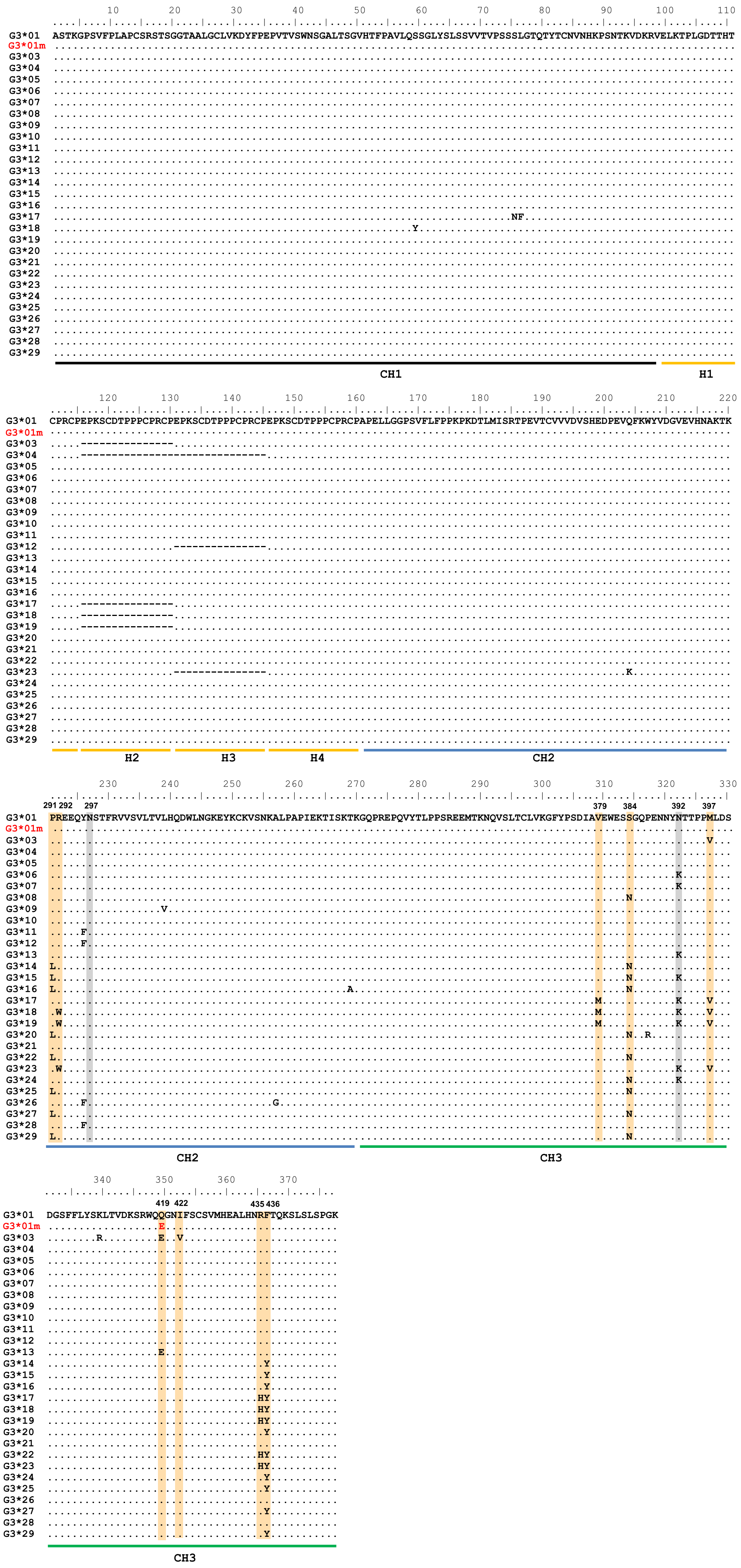

10 20 30 40 50 60 70 80 90 100 110
G3*01 ASTKGPSVFPLAPCSRSTSGGTAALGCLVKDYFPEPVTVSWNSGALTSGVHTFPAVLQSSGLYSLSSVVTVPSSSLGTQTYTCNVNHKPSNTKVDKRVELKTPLGDTTHT
G3*01m
G3*03
G3*04
G3*05
G3*06
G3*07
G3*08
G3*09
G3*10
G3*11
G3*12
G3*13
G3*14
G3*15
G3*16
G3*17 NF
G3*18 Y
G3*19
G3*20
G3*21
G3*22
G3*23
G3*24
G3*25
G3*26
G3*27
G3*28
G3*29
CH1
H1
120 130 140 150 160 170 180 190 200 210 220
G3*01 CPRCPEPKSCDTPPPCPRCPEPKSCDTPPPCPRCPEPKSCDTPPPCPRCPAPELLGGPSVFLFPPKPKDTLMISRTPEVTCVVVDVSHEDPEVQFKWYVDGVEVHNAKTK
G3*23 K
H2
H3
H4
CH2
230 240 250 260 270 280 290 300 310 320 330
291 292 297 379 384 392 397
G3*01 PREEQYNSTFRVVSVLTVLHQDWLNGKEYKCKVSNKALPAPIEKTISKTKGQPREPQVYTLPPSREEMTKNQVSLTCLVKGFYPSDIAVEWESSGQPENNYNTTPPMLDS
G3*03 V
G3*06 K
G3*07 K
G3*08 N
G3*09 V
G3*11 F
G3*12 F
G3*13 K
G3*14 L N
G3*15 L N K
G3*16 L A N
G3*17 M K V
G3*18 W M K V
G3*19 W M K V
G3*20 L N R
G3*22 L N
G3*23 W K V
G3*24 N K
G3*25 L N
G3*26 F G
G3*27 L N
G3*28 F
G3*29 L N
CH2
CH3
340 350 360 370
419 422 435 436
G3*01 DGSFFLYSKLTVDKSRWQQGNIFSCSVMHEALHNRFTQKSLSLSPGK
G3*01m E
G3*03 R E V
G3*13 E
G3*14 Y
G3*15 Y
G3*16 Y
G3*17 HY
G3*18 HY
G3*19 HY
G3*20 Y
G3*22 HY
G3*23 HY
G3*24 Y
G3*25 Y
G3*27 Y
G3*29 Y
CH3
